# Independent Associations of Vitamin D Levels with Visceral Fat and Metabolic Age in Adults: A Retrospective Cross-Sectional Study

**DOI:** 10.3390/metabo16080539

**Published:** 2026-07-31

**Authors:** Veysel Ağan

**Affiliations:** Pharmacy Services Program, Health Services Vocational School, Harran University, 63300 Şanlıurfa, Türkiye; aganveysel@gmail.com; Tel.: +90-505-737-08-10

**Keywords:** body composition, bioelectrical impedance analysis, adiposity, body fat percentage, biological aging

## Abstract

Background/Objectives: Vitamin D deficiency has been associated with obesity and adverse metabolic outcomes. This study aimed to evaluate the associations between serum vitamin D levels and body composition parameters, including visceral fat level, body fat percentage, body mass index, muscle mass, and metabolic age, and to determine whether these relationships were independent of age and sex. Methods: This retrospective cross-sectional study included 164 individuals aged 18–64 years who had serum vitamin D measurements and concurrent body composition analyses between February 2023 and May 2025. Body composition was assessed using bioelectrical impedance analysis (Tanita BC-418), and serum vitamin D levels were measured by electrochemiluminescence immunoassay. Correlations were assessed using Spearman’s rank correlation analysis, and multivariate linear regression analyses were performed after adjusting for age and sex. Results: Serum vitamin D levels showed significant negative correlations with visceral fat level (r = −0.272; *p* < 0.001), body fat percentage (r = −0.225; *p* = 0.004), body mass index (r = −0.282; *p* < 0.001), and metabolic age (r = −0.254; *p* = 0.001). No significant association was found between serum vitamin D levels and muscle mass (*p* = 0.409). In multivariate regression analysis, serum vitamin D levels remained independently associated with visceral fat level (*p* = 0.017), body fat percentage (*p* = 0.015), and metabolic age (*p* = 0.026), whereas the association with body mass index was no longer significant (*p* = 0.114). Conclusions: Low vitamin D levels were independently associated with increased visceral adiposity, higher body fat percentage, and advanced metabolic age. Across all multivariable regression models, age consistently emerged as the strongest predictor, while vitamin D levels remained independently associated with visceral fat, body fat percentage, and metabolic age.

## 1. Introduction

Vitamin D, recognized for its critical roles in calcium-phosphorus homeostasis and skeletal mineralization, has been shown to participate in a broad range of biological processes—including immune modulation, inflammation control, cellular proliferation, and energy mechanisms—in addition to its pleiotropic properties [1]. Circulating vitamin D levels are considered the primary indicator of the body’s vitamin D status due to its status as the predominant metabolite in systemic circulation, its long biological half-life, and the fact that it is primarily synthesized in the liver [2].

Current evidence suggests that serum vitamin D deficiency is not merely a problem affecting bone structure, but may also play a role in the development of cardiometabolic disorders, immune dysfunction, and chronic inflammation [3,4]. Recent studies indicate a significant association between serum vitamin D concentrations and body composition parameters [5]. The sequestration of vitamin D within adipose tissue is considered a key mechanism leading to reduced serum levels and diminished biological activity of the vitamin, particularly in individuals with high body fat [6]. However, the reported associations between vitamin D and body composition parameters remain inconsistent across previous studies [7]. Vitamin D is closely associated with insulin sensitivity and may improve glucose metabolism by reducing pro-inflammatory cytokines and enhancing insulin receptor expression and insulin signaling [8]. The multifaceted nature of diseases associated with serum vitamin D deficiency necessitates a deeper understanding of this biomarker’s functional role in the biological aging process. Current biomarkers used to determine biological age serve as strategic tools for objectifying this process and predicting the socio-economic impacts of aging dynamics [9].

One of the modern parameters used to assess biological aging is “metabolic age,” a device-derived index based on comparing an individual’s basal metabolic rate (BMR) with the average chronological age of the same age group. Beyond being merely a chronological number, metabolic age is considered a functional reflection of the balance between body composition and basal energy expenditure [10]. The literature reports that data obtained via bioelectrical impedance analysis (BIA) may correlate with epigenetic aging biomarkers and markers of systemic inflammation, and may serve as a complementary tool for the early prediction of metabolic risk [11]. In particular, it has been suggested that pleiotropic micronutrients, such as vitamin D, modulate this “cellular tempo” through their roles in mitochondrial function and oxidative stress management [12,13].

Although previous studies have reported associations between vitamin D status and obesity-related parameters, most have primarily focused on conventional anthropometric indices such as BMI or general adiposity. To the best of our knowledge, few studies have simultaneously evaluated visceral fat, body fat percentage, muscle mass, BMI, and device-derived metabolic age within the same analytical framework while adjusting for important confounders such as age and sex in apparently healthy adults.

Based on the available evidence, we hypothesized that lower vitamin D levels would be independently associated with less favorable body composition parameters, particularly higher visceral fat and metabolic age, even after adjustment for age and sex. Moreover, the aim of this study was to investigate the independent associations between vitamin D levels and body composition parameters, including visceral fat, body fat percentage, muscle mass, BMI, and metabolic age, in apparently healthy adults.

In this context, the aim of this study was to evaluate the relationship between serum vitamin D levels and body composition parameters (body fat percentage, visceral fat levels, muscle mass, and metabolic age) in adults. Additionally, by examining whether this relationship varies according to body mass index (BMI), the study aimed to contribute to the understanding of the relationship between serum vitamin D levels and metabolic risk markers.

## 2. Materials and Methods

### 2.1. Study Design and Population

This study was designed as a retrospective, observational, and cross-sectional study. The study included adults aged 18–64 who presented to Harran University Hospital between 1 February 2023, and 31 May 2025, and for whom serum vitamin D levels and concurrent body composition measurements were available.

### 2.2. Data Collection

Demographic and biochemical data for the study were retrospectively obtained from the hospital information management system. Recorded variables included age, sex and body composition.

### 2.3. Body Composition Analysis

Body composition parameters were assessed using a segmental multi-frequency bioelectrical impedance analyzer (Tanita BC-418 MA, Tanita Corp., Tokyo, Japan). When compared to reference methods such as hydrostatic weighing, the Tanita BC-418 system has demonstrated acceptable validity and reliability in estimating body fat percentage and body composition parameters in adult populations. Additionally, the segmental 8-electrode configuration allows for regional and whole-body composition assessment in clinical and epidemiological settings [10,14]. The parameters obtained using this device included BMI, body fat percentage (%), muscle mass (kg), visceral fat level, and metabolic age.

### 2.4. Inclusion Criteria

A total of 164 apparently healthy adults were included in the study after screening the hospital database between 1 February 2023 and 31 May 2025. The participant selection process was based on predefined inclusion and exclusion criteria. Eligible participants were aged between 18 and 64 years and had concurrent serum vitamin D measurements and body composition analyses performed using the Tanita BC-418 MA device. Complete demographic, clinical, and biochemical records were required for inclusion. The final study population consisted of 65 males (39.6%) and 99 females (60.4%). The demographic and clinical characteristics of the study population are presented in Table 1.

Participants were excluded if they were pregnant or lactating; had active malignancy, chronic kidney disease (stage 4–5), advanced liver disease, uncontrolled thyroid dysfunction, acute infection, or inflammatory disease; or were receiving medications known to affect vitamin D metabolism, including corticosteroids, antiepileptic drugs, or high-dose vitamin D supplementation. Individuals with incomplete or unreliable records were also excluded.

These inclusion criteria were selected to establish a relatively homogeneous adult study population with complete biochemical and body composition data obtained during the same clinical visit. This approach was intended to minimize potential bias arising from missing or non-contemporaneous measurements and to improve the reliability of the analyses evaluating the associations between vitamin D levels and body composition parameters.

### 2.5. Ethical Approval

Ethical approval for this study was obtained from the Harran University Faculty of Medicine Non-Interventional Clinical Research Ethics Committee (Decision No: 2026-00071; Date: 18 May 2026). The study was conducted in accordance with the principles of the Declaration of Helsinki. Written informed consent was obtained from all participants prior to biochemical and body composition assessments.

### 2.6. Statistical Analysis

Statistical analyses were performed using SPSS (IBM SPSS Statistics, version 24.0) software. The normality of continuous variables was assessed using the Shapiro–Wilk test and confirmed using the Kolmogorov–Smirnov test. Data showing a normal distribution were expressed as mean ± standard deviation, while data not showing a normal distribution were expressed as median (minimum–maximum). Categorical variables were presented as counts and percentages (%).

Since serum vitamin D levels did not follow a normal distribution, non-parametric methods were preferred for correlation analyses. However, the distribution of standardized residuals was examined in multivariate regression analyses, and it was confirmed that the model assumptions were met.

Serum vitamin D levels were analyzed as a continuous variable. Since categorical classifications could lead to loss of information and there was a significant numerical imbalance between groups, the analyses were conducted using continuous variables.

The relationships between serum vitamin D levels and body composition parameters were evaluated using Spearman’s rank correlation analyses.

To assess the independent effect of serum vitamin D levels on body composition parameters, a multivariate linear regression analysis was performed after adjusting for age and sex. Multicollinearity was assessed using the variance inflation factor (VIF).

Although serum vitamin D values were not normally distributed, the assumptions of linear regression were evaluated based on the distribution of the standardized residuals rather than the distribution of serum vitamin D values themselves. Since the standardized residuals showed no substantial deviation from normality and the assumptions of linear regression were adequately satisfied, logarithmic transformation of serum vitamin D values was not considered necessary.

Serum vitamin D levels may vary depending on seasonal sun exposure and clothing style. However, data regarding the season in which blood samples were collected and the participants’ clothing/covering levels were not available in this study; therefore, seasonal and clothing-related adjustments could not be made. This limitation was taken into account in the interpretation of the results.

Metabolic age is a parameter derived by the device, calculated based on basal metabolic rate and body composition. Metabolic age was determined using the device’s (Tanita BC-418) proprietary algorithm, which compares the participants’ basal metabolic rate to the average basal metabolic rate values of their respective chronological age groups. This index is intended to reflect the body’s metabolic efficiency based on lean body mass and energy expenditure.

A statistical significance level of *p* < 0.05 was accepted.

A post hoc power analysis was conducted to determine the power of the tests at a significance level using G*Power (version 3.1; Heinrich Heine University Düsseldorf, Düsseldorf, Germany).

## 3. Results

A total of 164 individuals (65 men, 99 women) were included in the study. The mean age of the participants was 35.29 ± 13.52 (18–63) years. The mean serum vitamin D level was 11.59 ± 7.86 ng/mL. When body composition parameters were examined, the mean body fat percentage was 29.10 ± 9.10%, metabolic age was 38.45 ± 15.16 years, visceral fat level was 6.62 ± 4.39, and BMI was 27.31 ± 5.34 kg/m^2^. The mean muscle mass was determined to be 51.38 ± 11.04 kg. The demographic, clinical, and biochemical characteristics of the study population are summarized in Table 1.

According to the results of the Spearman correlation analysis, serum vitamin D levels showed a significant negative correlation with visceral fat levels (r = −0.272, *p* < 0.001). Similarly, a significant negative relationship was found with metabolic age (r = −0.254, *p* = 0.001). A significant negative relationship was also detected between BMI and serum vitamin D levels (r = −0.282, *p* < 0.001). Additionally, a similar negative and significant correlation was observed with body fat percentage (%) (r = −0.225, *p* = 0.004).

In contrast, no statistically significant association was found between muscle mass and serum vitamin D levels (r = −0.065, *p* = 0.409). However, when body composition parameters were evaluated in relation to one another, strong positive associations were observed. In particular, a very strong positive correlation was found between visceral fat levels and metabolic age (r = 0.888, *p* < 0.001). Similarly, a strong positive relationship was found between visceral fat level and BMI (r = 0.793, *p* < 0.001), and a strong, positive, and statistically significant relationship was reported between body fat percentage and BMI (r = 0.634, *p* < 0.001).

When muscle mass was evaluated, a significant positive correlation was found with visceral fat levels (r = 0.542, *p* < 0.001) and a positive correlation with BMI (r = 0.462, *p* < 0.001). In contrast, a significant negative correlation was found between muscle mass and body fat percentage (r = −0.272, *p* < 0.001). Additionally, a weak but significant positive correlation was found between muscle mass and metabolic age (r = 0.274, *p* < 0.001).

**Figure 1:**
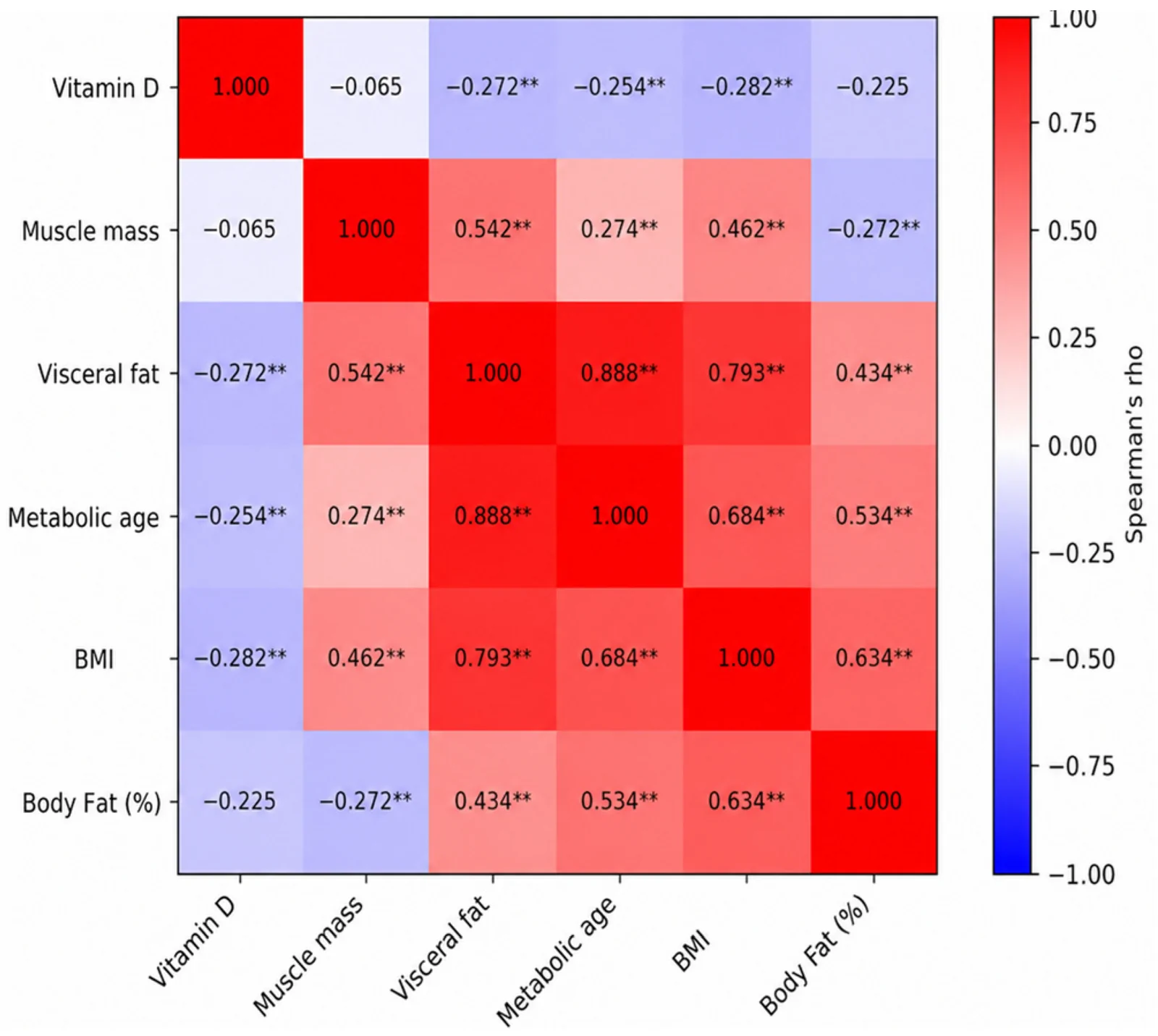
Heatmap of Spearman correlation coefficients between serum vitamin D levels and body composition parameters. ** indicates statistical significance at *p* < 0.01.

Spearman correlation analyses between serum vitamin D levels and body composition parameters are presented in Table 2. Additionally, the correlation heatmap is shown in Figure 1.

The heatmap illustrates the strength and direction of correlations using a blue–white–red color scale. Blue indicates negative correlations, red indicates positive correlations, and white represents no correlation. Values are Spearman’s rho coefficients. Statistically significant correlations (*p* < 0.01) are marked with ** in Figure 1.

In this study, serum vitamin D levels and age were included as continuous variables, while sex was included as a categorical variable (male = 1, female = 2) in the multivariate linear regression analysis. Visceral fat level, BMI, body fat percentage (%), and metabolic age were evaluated as dependent variables. In the multivariate regression analyses, the independent effects of serum vitamin D levels, age, and sex on these body composition parameters were examined.

The variables used in the regression analysis in this study:

Dependent variables:

- Visceral Fat
- BMI
- Body Fat Percentage
- Metabolic Age

Independent variables:

- Vitamin D
- Age
- Sex

According to the results of the regression analysis,

Regression Results (Model 1—Visceral Fat)

According to the results of the multivariate linear regression analysis, serum vitamin D levels, age, and sex were included in the model.

The model was found to be statistically significant (F = 88.738; *p* < 0.001) and explained 62.5% of the variance in visceral fat levels (R^2^ = 0.625; Adj. R^2^ = 0.618). The model’s error structure was confirmed to be appropriate by the Durbin-Watson value (DW = 2.041), and no autocorrelation was observed.

The model results indicate that serum vitamin D levels have a significant, independent, and negative effect on visceral fat levels (B = −0.066; β = −0.118; t = −2.409; *p* = 0.017). Lower vitamin D levels are associated with higher visceral fat accumulation.

The age variable was found to be the strongest independent predictor of visceral fat levels (B = 0.225; β = 0.692; t = 13.947; *p* < 0.001). Age was positively associated with visceral fat levels.

The sex variable also had a significant effect on visceral fat levels (B = −2.175; β = −0.243; t = −4.974; *p* < 0.001). Female sex was significantly associated with lower visceral fat levels compared to male sex. Linear Regression Line y = 2.949 − 0.066x R^2^ = 0.055.

**Figure 2:**
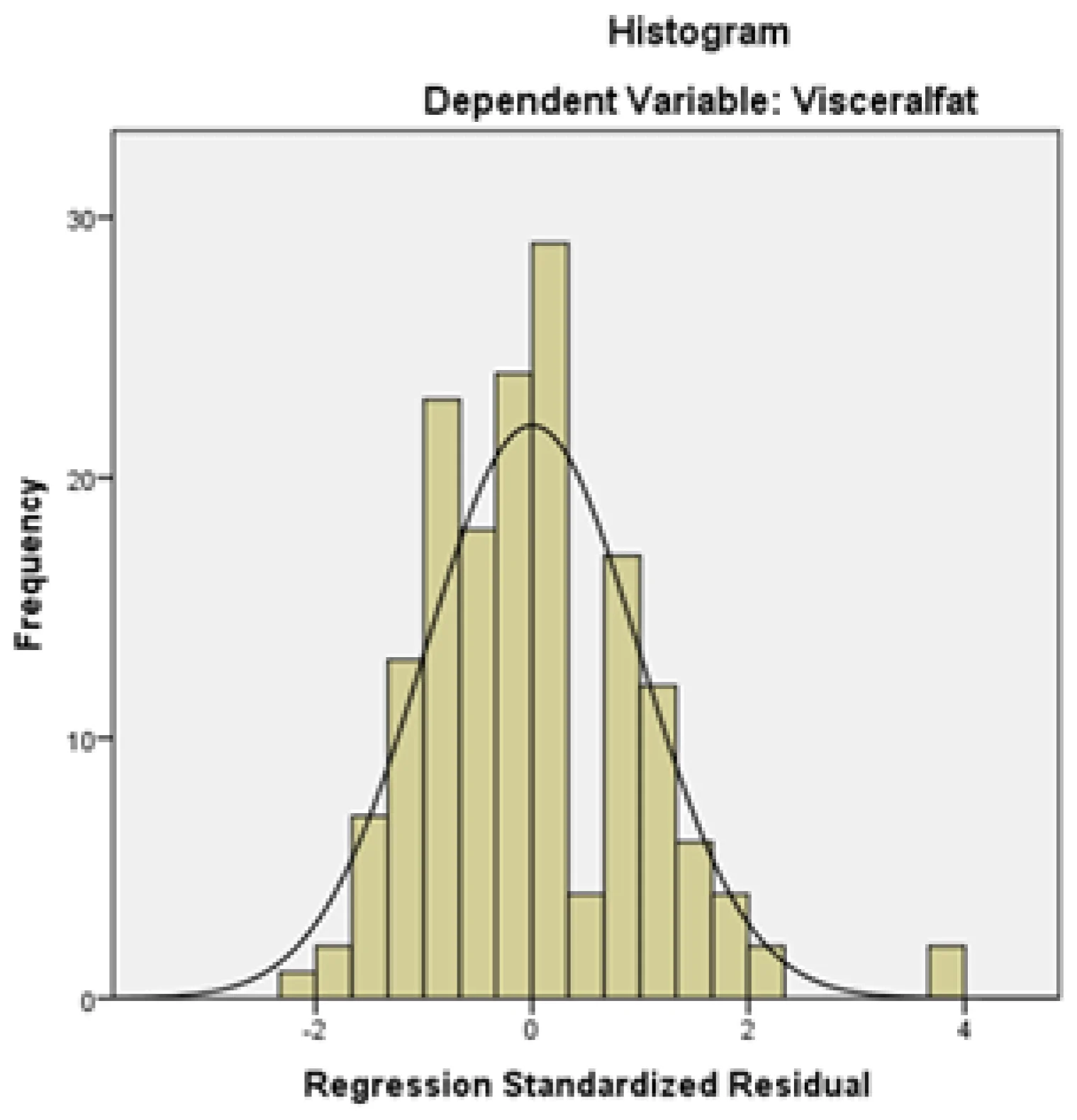
Histogram of standardized residuals for the regression model of visceral fat.

**Figure 3:**
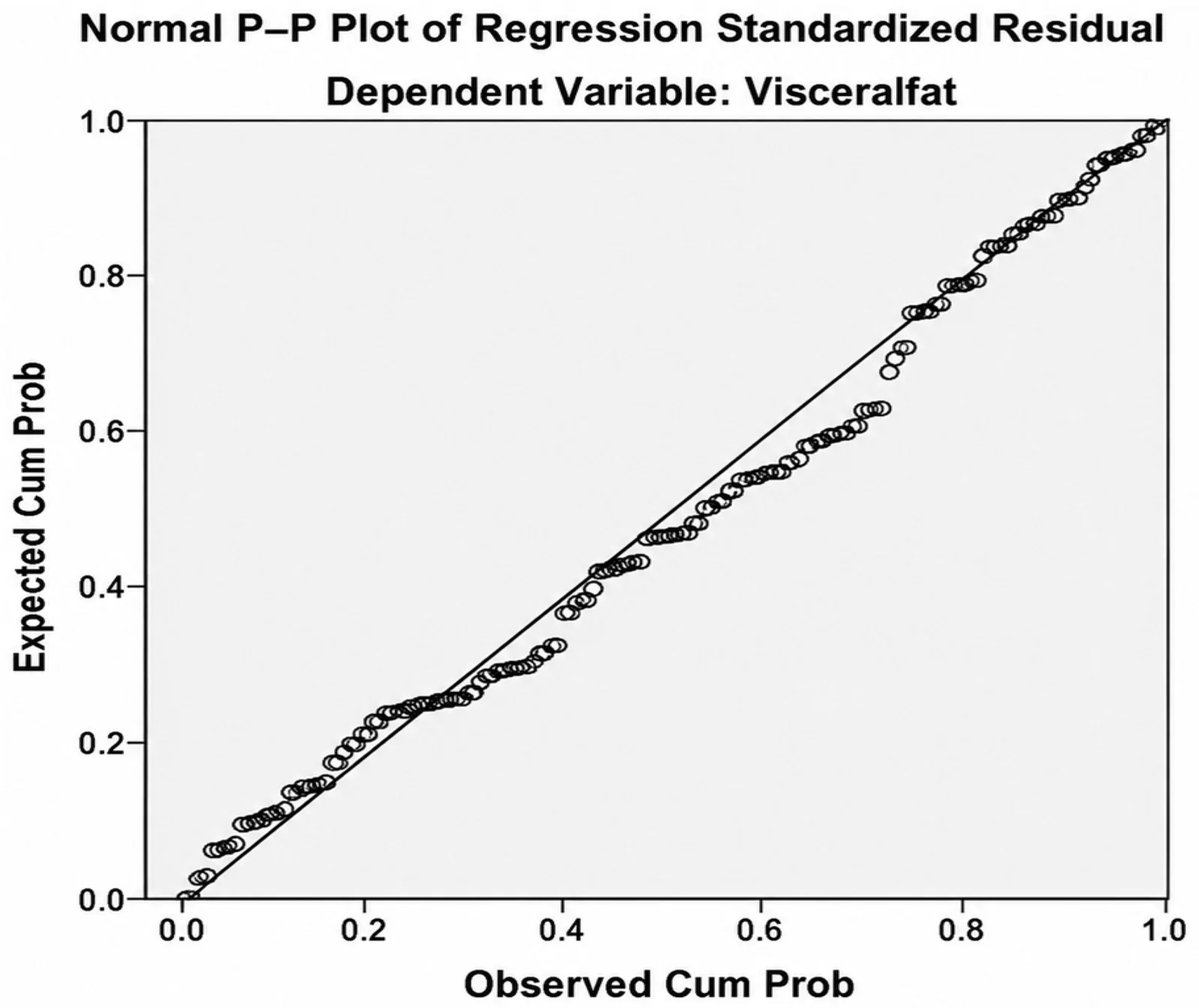
Normal P–P plot of regression standardized residuals for the visceral fat regression model.

**Figure 4:**
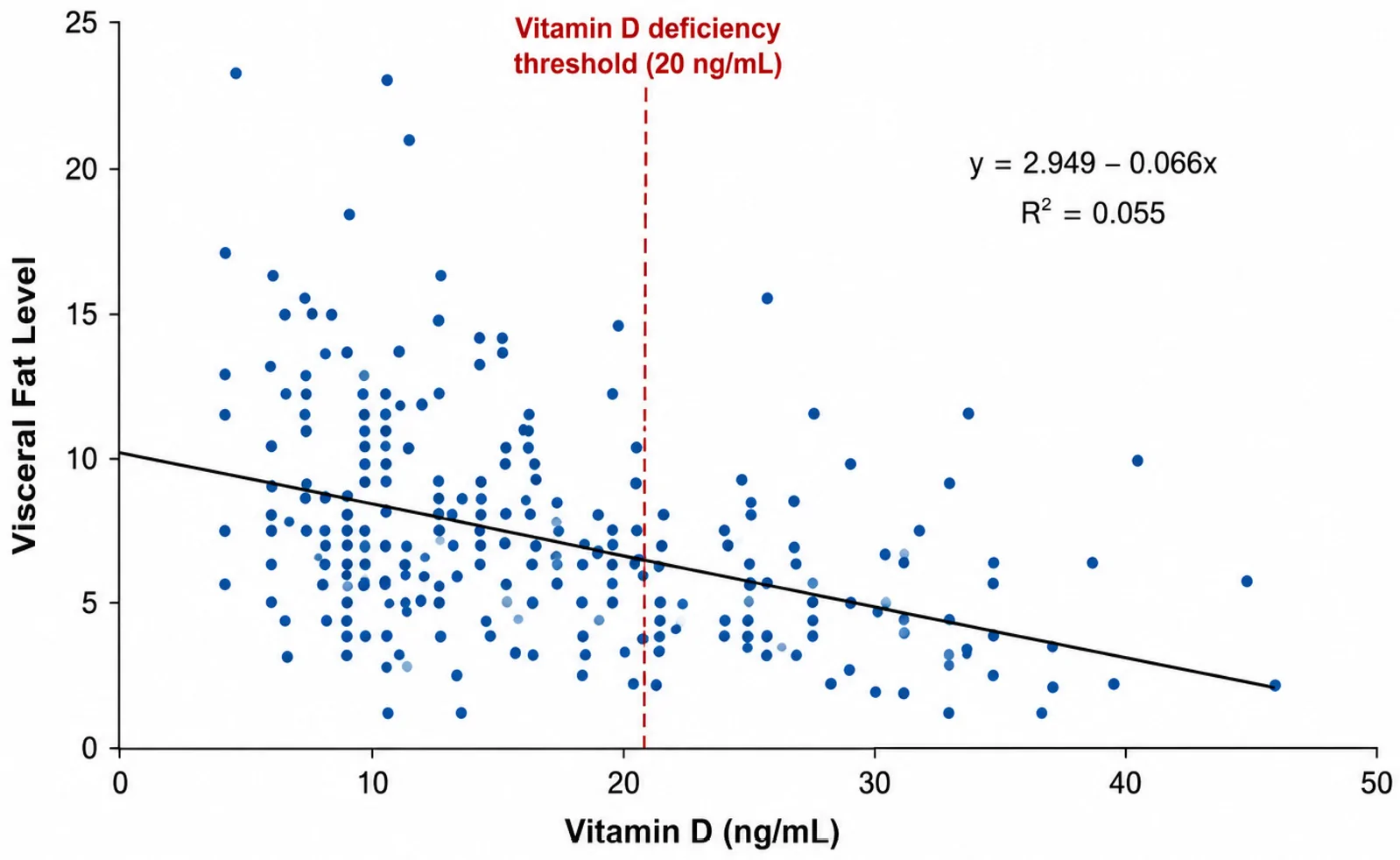
Scatter plot demonstrating the inverse association between Vitamin D levels and visceral fat.

Figure 2: Histogram of standardized residuals for the regression model of visceral fat; Figure 3: Normal P–P plot of regression standardized residuals for the visceral fat regression model; and Figure 4: Scatter plot demonstrating the inverse association between serum vitamin D levels and visceral fat are shown as graphical findings of the study.

Regression Results (Model 2—BMI)

In the multiple linear regression analysis, serum vitamin D levels, age, and sex were included in the model together.

The model was found to be statistically significant (F = 15.063; *p* < 0.001). However, the model’s explanatory power is moderate, accounting for 22% of the variance in BMI (R^2^ = 0.220; Adj. R^2^ = 0.206). The model’s error structure is appropriate, with a Durbin-Watson value of 2.031, indicating no autocorrelation.

According to the analysis results, it was determined that serum vitamin D levels have no independent effect on BMI (B = −0.077; β = −0.113; t = −1.590; *p* = 0.114).

In contrast, it was determined that the age variable has a significant and positive independent effect on BMI (B = 0.174; β = 0.440; t = 6.157; *p* < 0.001). This finding indicates that body mass index increases with age.

No statistically significant relationship was found between the sex variable and BMI (*p* = 0.538).

Regression Results (Model 3—Body Fat Percentage)

In the multivariate linear regression analysis, serum vitamin D levels, age, and sex were included in the model together.

The model was found to be statistically significant (F = 52.777; *p* < 0.001) and explains 49.7% of the variance in body fat percentage (R^2^ = 0.497; Adj. R^2^ = 0.488). The model’s error structure was appropriate, with a Durbin-Watson value of 1.968.

It was determined that serum vitamin D levels have an independent and significantly negative effect on body fat percentage (B = −0.162; β = −0.140; t = −2.460; *p* = 0.015). This finding indicates that lower vitamin D levels are associated with a higher body fat percentage.

It was found that the age variable has a positive and significant effect on body fat percentage (B = 0.228; β = 0.339; t = 5.896; *p* < 0.001). Body fat percentage increases with age.

The sex variable was found to be the strongest predictor of body fat percentage (B = 11.774; β = 0.635; t = 11.222; *p* < 0.001). A significantly higher body fat percentage was observed in women compared to men.

Regression Findings (Model 4—Metabolic Age)

In the multiple linear regression analysis, serum vitamin D levels, age, and sex were included in the model together.

The model was found to be statistically significant (F = 389.157; *p* < 0.001) and explains 87.9% of the variance in the metabolic age variable (R^2^ = 0.879; Adj. R^2^ = 0.877). The model’s error structure is appropriate, with a Durbin-Watson value of 1.964.

The age variable was identified as the strongest predictor of metabolic age (B = 1.046; β = 0.933; t = 33.184; *p* < 0.001). This finding indicates that metabolic age increases significantly as chronological age increases.

It was determined that the sex variable has a small but statistically significant effect on metabolic age (B = 2.990; β = 0.097; *p* = 0.001).

It was found that serum vitamin D levels have a weak but independent and significant negative association with metabolic age (B = −0.121; β = −0.063; t = −2.243; *p* = 0.026). Higher vitamin D levels are associated with lower metabolic age.

Linear Regression Line y = 45 − 0.48x R^2^ = 0.049. A 1 ng/mL increase in vitamin D levels is associated with an approximate 0.48-unit decrease in metabolic age.

**Figure 5:**
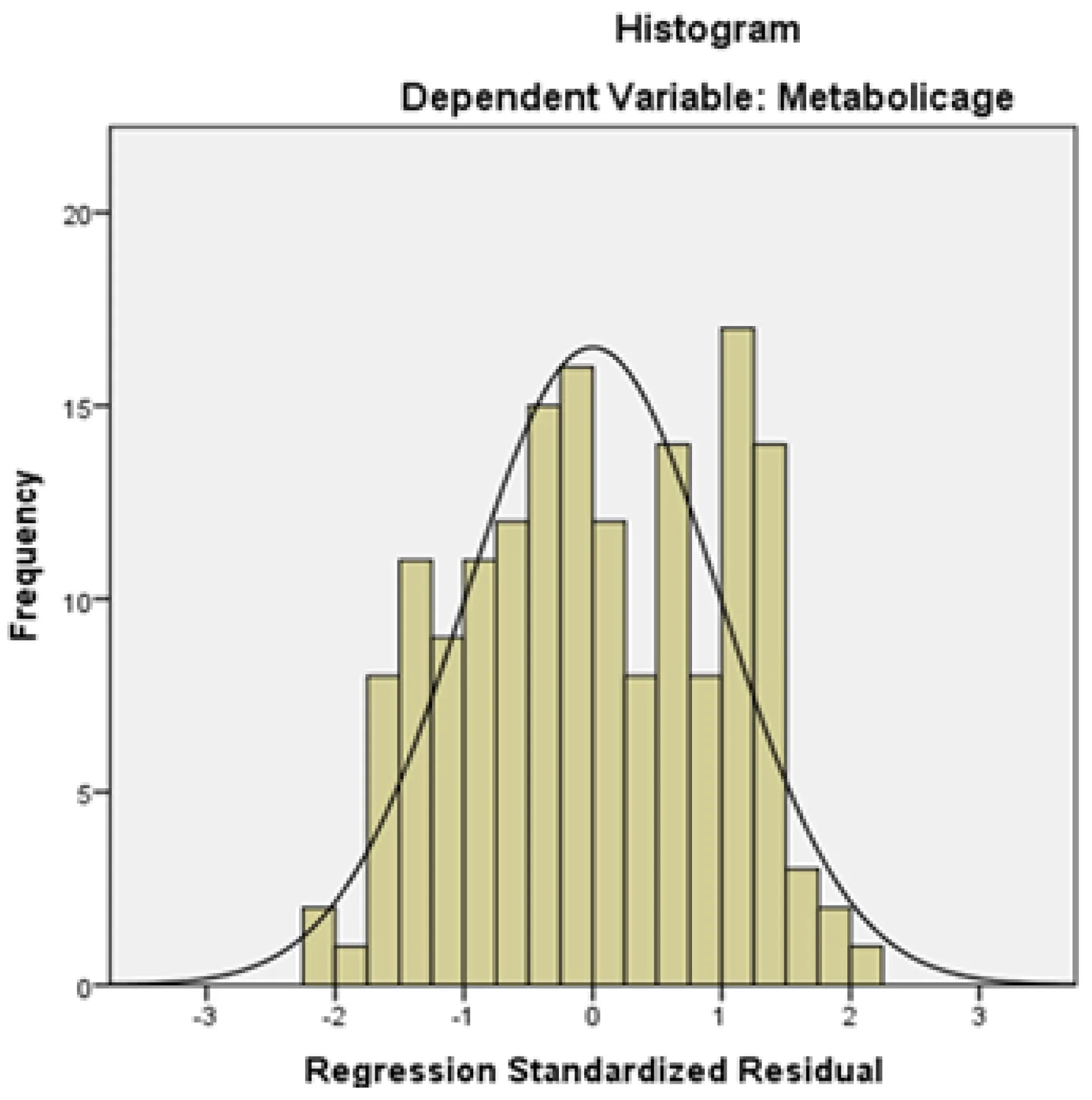
Histogram of standardized residuals for the regression model of metabolic age.

**Figure 6:**
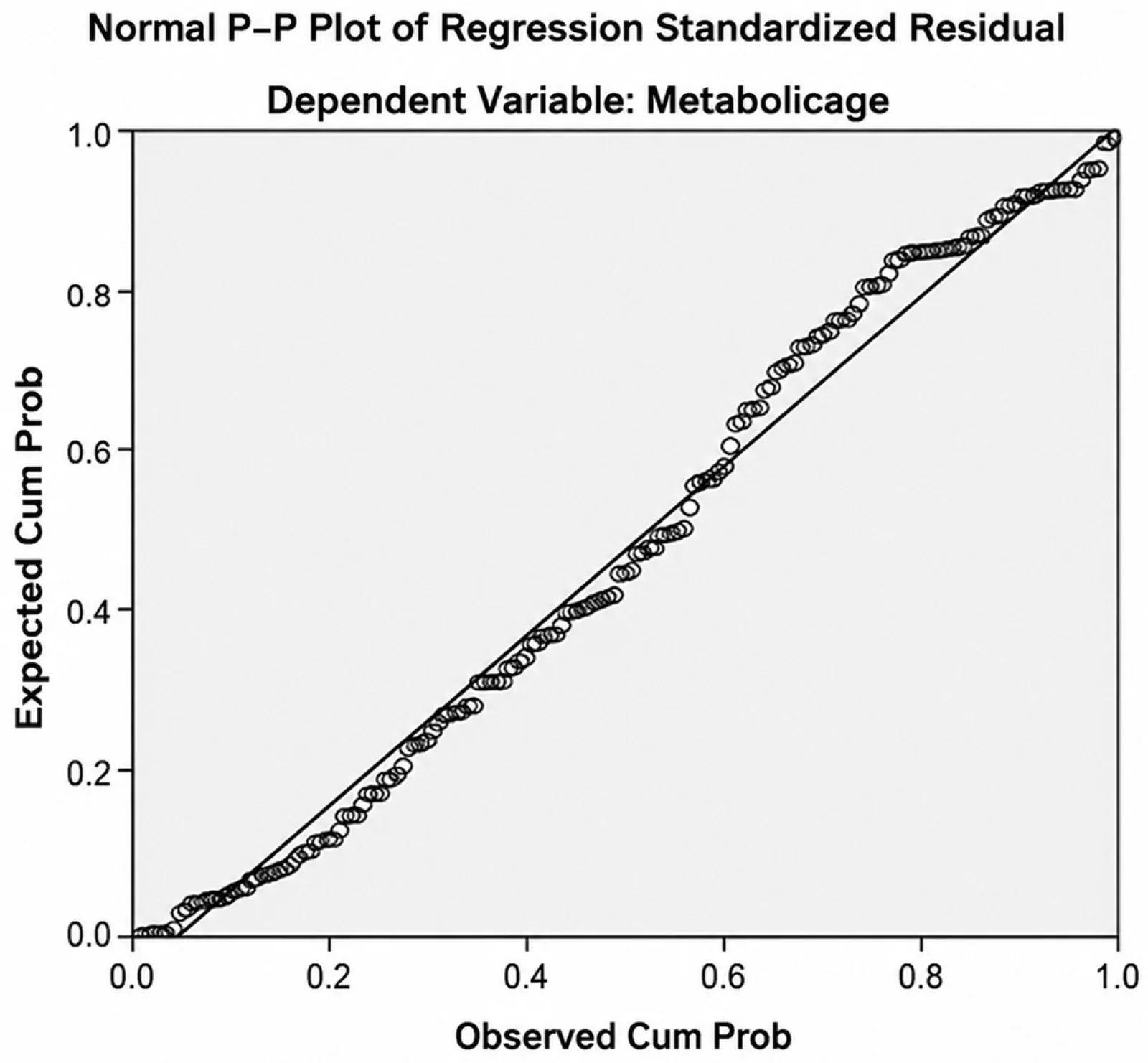
Normal P–P plot of regression standardized residuals for the metabolic age regression model.

**Figure 7:**
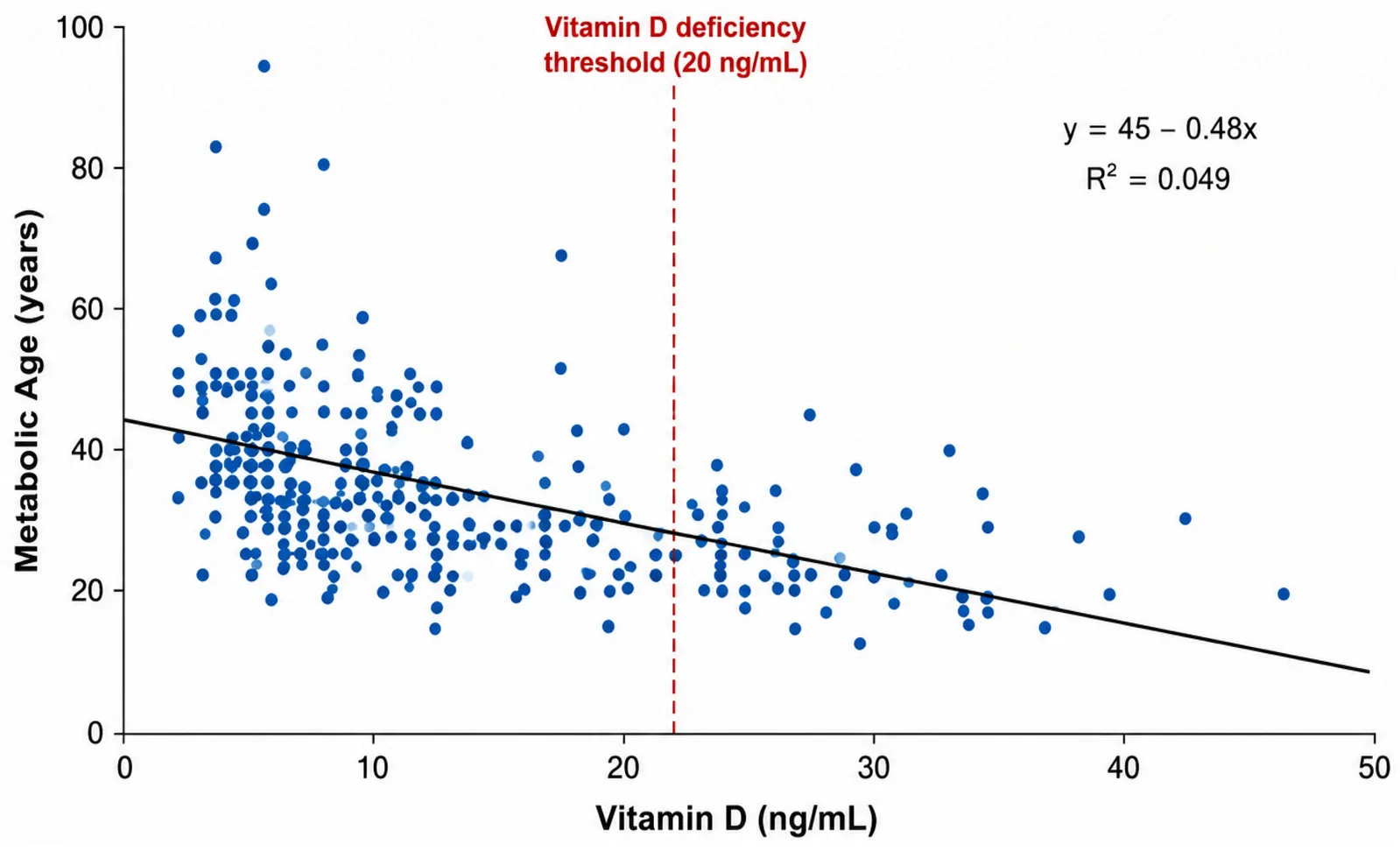
Scatter plot demonstrating the inverse association between serum vitamin D levels and metabolic age.

Figure 5:. Histogram of standardized residuals for the regression model of metabolic age, Figure 6: Normal P–P plot of regression standardized residuals for the metabolic age regression model, and Figure 7: Scatter plot demonstrating the inverse association between serum vitamin D levels and metabolic age are shown as graphical findings of the study.

The results of the multiple linear regression analysis between serum vitamin D levels and body composition parameters identified in the study are presented in Table 3.

No multicollinearity was detected in any of the four separate multivariate linear regression models (all VIF values < 1.1). These findings indicate that there is no significant linear relationship among the independent variables and that the model was statistically reliable. In all models: serum vitamin D → VIF ≈ 1.03; Age → VIF ≈ 1.04; Sex → VIF ≈ 1.02.

Based on the observed effect size (r ≈ 0.27), an alpha level of 0.05, and a sample size of 164 participants, the statistical power of the study was calculated to be approximately 0.85, indicating adequate power to detect significant associations.

## 4. Discussion

In this study, the relationship between serum vitamin D levels and body composition parameters in adults was comprehensively evaluated. The findings revealed that serum vitamin D levels were significantly and negatively associated with visceral fat levels, body fat percentage, body mass index, and metabolic age. Multivariate regression analyses showed that this relationship persisted, particularly regarding visceral fat and body fat percentage, independent of age and sex. In contrast, no significant association was found between muscle mass and serum vitamin D levels. Additionally, age emerged as the strongest determinant, particularly for visceral fat and metabolic parameters.

One of the major limitations of this study is that it did not account for seasonal variability, which can influence serum vitamin D levels. In addition to seasonal differences in sun exposure, individuals’ clothing choices and level of coverage can also significantly affect vitamin D synthesis. However, since the study lacked data on the season in which blood samples were collected and on participants’ clothing/covering characteristics, these factors could not be included in the analysis. This should be taken into account when interpreting the results [15,16].

The current literature indicates that the relationship between serum vitamin D levels and visceral adiposity is generally inverse. Epidemiological studies conducted in various populations have demonstrated that increased visceral fat tissue is associated with lower vitamin D levels. Indeed, large-scale studies have reported significant negative correlations between visceral adipose tissue thickness and serum vitamin D concentrations, suggesting that visceral obesity may be an independent risk factor for vitamin D deficiency [17,18]. Similarly, a study conducted in individuals with type 2 diabetes demonstrated that the prevalence of visceral fat accumulation was significantly higher in groups with vitamin D insufficiency and deficiency [19]. These findings support the conclusion that the negative association between serum vitamin D levels and visceral fat levels observed in our study is consistent with the literature.

However, some studies have reported that this association is not consistent across all populations. In particular, no significant association between vitamin D and visceral adipose tissue has been observed in conditions where physiologically distinct metabolic processes predominate, such as pregnancy [20]. This suggests that the vitamin D–adipose tissue interaction may be influenced by factors such as age, hormonal status, and metabolic profile. Furthermore, some studies suggest that the relationship with vitamin D may be more pronounced because visceral adipose tissue is metabolically more active compared to subcutaneous adipose tissue [21]. The independent association between vitamin D levels and visceral adiposity remained significant after adjustment for age and sex. In this context, the fact that our study has significantly demonstrated an independent and negative relationship between visceral fat levels and serum vitamin D can be considered an important finding supporting the notion that vitamin D may interact more strongly with metabolically active fat deposits.

The biological mechanisms underlying the inverse relationship between serum vitamin D levels and visceral adiposity are multifaceted; the most commonly proposed explanations include the sequestration of vitamin D in adipose tissue, a volumetric dilution effect, and chronic low-grade inflammation. Because vitamin D is a lipophilic molecule, it tends to be stored primarily in visceral and subcutaneous adipose tissue. This phenomenon is explained by a “sequestration” mechanism that leads to decreased circulating vitamin D levels in individuals with increased fat mass [22]. The reduced bioavailability of vitamin D retained in adipose tissue may cause serum levels to fail to reflect true physiological reserves.

In addition, the “volumetric dilution hypothesis” has emerged as an important mechanism explaining low serum vitamin D levels [23]. According to this approach, as total body volume and particularly fat mass increase, circulating vitamin D is distributed across a larger volume, leading to relatively low measured serum concentrations [24]. Accordingly, low serum vitamin D levels in obese individuals may reflect a distributional effect rather than an absolute deficiency.

The relationship between vitamin D status and adiposity is likely to be bidirectional. On the one hand, increased adipose tissue may reduce circulating vitamin D concentrations through sequestration within fat tissue and volumetric dilution. On the other hand, experimental studies suggest that vitamin D deficiency may influence adipocyte differentiation, lipid metabolism, and inflammatory pathways, potentially promoting adipose tissue accumulation. Therefore, both mechanisms may contribute to the inverse association observed between vitamin D levels and visceral adiposity. However, because the present study employed a cross-sectional design, the direction of this relationship cannot be determined, and causal inferences should be made with caution.

Another important mechanism is chronic low-grade inflammation, which is closely associated with obesity. Visceral adipose tissue, in particular, is a metabolically active organ that actively secretes proinflammatory cytokines (e.g., TNF-α, IL-6). This inflammatory environment can both affect vitamin D metabolism and modulate its effects in target tissues via vitamin D receptor expression. It has also been suggested that inflammation may influence the conversion of vitamin D to its active form in the liver and kidneys [25]. In this context, the inflammatory state associated with increased visceral fat suggests a bidirectional relationship in which low serum vitamin D levels may be both a cause and a consequence.

One of the notable findings in our study is that while a significant correlation was detected between serum vitamin D levels and BMI in the correlation analysis, this relationship lost its independence in the multivariate regression model. This can be explained by the limitations of BMI in reflecting body composition. BMI is a crude measure that normalizes total body weight relative to height and cannot distinguish between fat mass and lean mass. Moreover, it may yield misleading results, particularly in individuals with high muscle mass, and fails to fully reflect true adiposity [26,27].

In contrast, more specific composition parameters, such as visceral fat levels and body fat percentage, are measures more directly associated with metabolic risk. Indeed, the fact that serum vitamin D levels maintained an independent association with these parameters in our study suggests that vitamin D-related metabolic effects are more closely linked to the distribution and quality of adipose tissue rather than overall body size [28,29]. The fact that BMI loses its significance in the regression model indicates that this variable conveys information that overlaps with stronger predictors, such as visceral fat and total body fat percentage, and that its independent contribution within the model is therefore limited [30].

Furthermore, the fact that the age variable has a strong and independent effect on BMI suggests that BMI largely reflects age-related changes in body composition dynamics. In this context, the increase in fat mass and decrease in muscle mass that accompany aging are indirectly represented through BMI; however, this measure is insufficient for distinguishing qualitative changes in body composition [31]. This suggests that the relationship between vitamin D and BMI may be indirect and mediated rather than direct.

The findings indicate that BMI is not a sufficient indicator on its own for evaluating the relationship with vitamin D levels, and instead, more specific parameters such as visceral fat and body fat percentage should be preferred. This approach emphasizes the importance of evaluating body composition using more precise measures in studies [32,33].

In our study, no statistically significant association was found between serum vitamin D levels and muscle mass. This finding is consistent with the heterogeneous results in the literature and suggests that the effects of vitamin D on the musculoskeletal system may be more closely related to muscle function. Indeed, many studies have shown that vitamin D levels are more strongly associated with muscle strength, performance, and physical function rather than muscle mass [34,35]. This supports the notion that the effects of vitamin D on muscle tissue occur through neuromuscular function and muscle contractility rather than direct hypertrophy.

Furthermore, muscle mass and other body composition parameters were assessed using direct segmental multifrequency bioelectrical impedance analysis (BIA). Although dual-energy X-ray absorptiometry (DEXA) is generally regarded as a reference method for body composition assessment, BIA represents a practical and well-validated alternative for clinical and epidemiological studies. It is non-invasive, radiation-free, rapid, and relatively inexpensive, making it suitable for routine assessments in large study populations. Previous validation studies have demonstrated acceptable agreement between the Tanita BC-418 and reference methods for estimating body composition. Nevertheless, because BIA measurements may be influenced by hydration status and other physiological factors, the use of DEXA or other reference techniques in future studies may further improve the precision of body composition assessment [36,37].

Furthermore, the finding of a positive association between muscle mass and visceral fat levels as well as BMI in our study suggests that muscle mass alone may not reflect a healthy metabolic status. Particularly in overweight or obese individuals, a “masked” condition may exist where an increase in fat mass occurs even if total muscle mass is high. In this context, it can be argued that assessing the relationship between vitamin D and muscle mass independently of other components of body composition is challenging [33,38].

The cross-sectional design of our study limits our ability to establish potential temporal and causal relationships between vitamin D levels and muscle mass. While it has been suggested that vitamin D deficiency may lead to muscle mass loss in the long term, it has been reported that this effect becomes more pronounced, particularly in older age groups and in the context of sarcopenia development. The relatively young average age of our study population may also have played a role in the failure to detect this relationship [34,39].

The negative and independent association observed in our study between serum vitamin D levels and metabolic age is a notable and potentially novel finding, given that it has been addressed in only a limited number of studies in the current literature. Metabolic age is a composite indicator calculated based on an individual’s basal metabolic rate and body composition parameters, and is considered to reflect biological aging. In this context, the finding that higher vitamin D levels are associated with a lower metabolic age suggests that vitamin D may also be related to systemic aging processes [40,41].

When examining the potential mechanisms underlying this relationship, processes such as the anti-inflammatory effects of vitamin D, the reduction in oxidative stress, and the regulation of mitochondrial function may help explain this association. Chronic low-grade inflammation and oxidative stress are among the key determinants of biological aging, and vitamin D’s potential to modulate these processes may explain its effect on metabolic age. Additionally, vitamin D’s positive effects on insulin sensitivity and its role in regulating energy metabolism may be directly related to the components of metabolic age [42,43].

Vitamin D has also been implicated in maintaining mitochondrial function through its effects on calcium homeostasis, oxidative phosphorylation, and the regulation of reactive oxygen species. Improved mitochondrial efficiency may contribute to more effective cellular energy metabolism and maintenance of basal metabolic function. Although the metabolic age estimate provided by the Tanita BC-418 is derived from a proprietary algorithm that is not publicly available, it incorporates basal metabolic rate and body composition parameters. Therefore, the observed association between vitamin D status and metabolic age in the present study may reflect biologically plausible relationships rather than a direct mechanistic effect. Further studies using validated biological aging markers are needed to better clarify this association.

The limited number of studies in the literature examining the relationship between vitamin D and epigenetic aging and biological age markers suggests that vitamin D levels may be associated with slower aging processes [44]. In this context, our findings support the existing evidence regarding the potential effects of vitamin D on biological aging and provide an important foundation for future studies in this field.

The findings suggest that serum vitamin D levels may serve as a meaningful biomarker in the context of body composition and metabolic risk markers. In particular, the independent and negative associations observed with visceral fat and body fat percentage suggest that vitamin D levels could be used as a complementary parameter in the assessment of cardiometabolic risk [17,45].

Furthermore, it highlights the importance of monitoring vitamin D levels and planning appropriate replacement strategies when necessary, particularly in individuals at high risk for the development of obesity and metabolic syndrome. However, the literature contains conflicting results regarding the effects of vitamin D supplementation on body composition, and randomized controlled trials in this area will provide stronger evidence [46]. In addition, future longitudinal cohort studies incorporating repeated seasonal measurements of serum vitamin D levels, together with well-designed vitamin D supplementation trials, are needed to clarify the temporal relationships and potential causal effects of vitamin D on body composition and metabolic health.

One of the most significant strengths of this study is that the relationship between serum vitamin D levels and body composition parameters was evaluated not only through simple correlation analyses but also using multivariate regression models adjusted for potential confounding variables such as age and sex. This approach supports the independence of the findings, thereby strengthening the reliability of the results. Furthermore, the inclusion of parameters such as visceral fat levels, body fat percentage, and metabolic age—which are more specific than BMI—in the analyses ensures that the study provides a more in-depth perspective regarding metabolic risk assessment. In addition, the examination of the relationship between metabolic age and vitamin D levels contributes to this topic, which has been addressed in only a limited number of studies in the literature, thereby adding to the existing literature.

Future experimental studies, including in vitro and in vivo investigations, are warranted to further clarify the biological mechanisms linking vitamin D status with body composition and metabolic aging.

## 5. Conclusions

In conclusion, this study demonstrated significant and multidimensional associations between vitamin D levels and body composition parameters. In addition, age consistently emerged as the strongest predictor in the multivariable regression analyses, highlighting the importance of considering age when evaluating the associations between vitamin D status and body composition parameters. In particular, the independent and negative relationships observed with visceral fat, body fat percentage, and metabolic age support a potential role of vitamin D. Conversely, the fact that BMI did not emerge as an independent predictor underscores the need for more specific parameters in assessing body composition. These findings suggest that vitamin D status may be considered as part of comprehensive metabolic risk assessment. However, prospective longitudinal studies incorporating seasonal assessment of vitamin D status, as well as large-scale randomized controlled supplementation trials, are needed to establish the temporal and causal nature of these relationships.

## Figures and Tables

**Table 1:** Baseline Characteristics of the Study Population.

Variable	Total (*n* = 164)
Age (years)	35.29 ± 13.52
Sex, *n* (%)	
Male	65 (39.6)
Female	99 (60.4)
Serum Vitamin D (ng/mL)	11.59 ± 7.86
Body Mass Index (kg/m^2^)	27.31 ± 5.34
Body Fat Percentage (%)	29.10 ± 9.10
Muscle Mass (kg)	51.38 ± 11.04
Visceral Fat Level	6.62 ± 4.39
Metabolic Age (years)	38.45 ± 15.16

Data are presented as mean ± standard deviation (SD) unless otherwise indicated. Categorical variables are presented as number (percentage).

**Table 2:** Spearman correlations between vitamin D and body composition parameters.

**Parameters**		**Muscle Mass**	**Visceral Fat**	**Metabolic Age**	**BMI (kg/m^2^)**	**Body Fat (%)**
Vitamin D	rho *p*	−0.065 0.409	−0.272 ** <0.001	−0.254 ** 0.001	−0.282 ** <0.001	−0.225 ** 0.004
Muscle mass	rho *p*		0.542 ** <0.001	0.274 ** <0.001	0.462 ** <0.001	−0.272 ** <0.001
Visceral fat	rho *p*			0.888 ** <0.001	0.793 ** <0.001	0.434 ** <0.001
Metabolic age	rho *p*				0.684 ** <0.001	0.534 ** <0.001
BMI (kg/m^2^)	rho *p*					0.634 ** <0.001

** Correlation is significant at the 0.01 level.

**Table 3:** Independent associations between serum vitamin D and body composition parameters (multiple linear regression analysis).

Dependent Variable	Predictor	B (95% CI)	β	*p*	VIF
Visceral Fat (R^2^ = 0.625, Adj. R^2^ = 0.618)					
	sex	−2.18	−0.243	<0.001	1.02
	age	0.23	0.692	<0.001	1.05
	vitamin D	−0.07	−0.118	0.017	1.03
BMI (kg/m^2^) (R^2^ = 0.220, Adj. R^2^ = 0.206)					
	sex	0.47	0.044	0.538	1.02
	age	0.17	0.440	<0.001	1.05
	vitamin D	−0.08	−0.113	0.114	1.03
Body Fat (%) (R^2^ = 0.497, Adj. R^2^ = 0.488)					
	sex	11.77	0.635	<0.001	1.02
	age	0.23	0.339	<0.001	1.05
	vitamin D	−0.16	−0.140	0.015	1.03
Metabolic Age (R^2^ = 0.879, Adj. R^2^ = 0.877)					
	sex	2.99	0.097	0.001	1.02
	age	1.05	0.933	<0.001	1.05
	vitamin D	−0.12	−0.063	0.026	1.03

## Data Availability

All data supporting the findings of this study are available within the paper. Other clinical and biochemical datasets used in this study are available from the corresponding author upon reasonable request, subject to compliance with institutional data protection regulations and ethical approval.
